# Solution NMR and molecular dynamics reveal a persistent alpha helix within the dynamic region of PsbQ from photosystem II of higher plants

**DOI:** 10.1002/prot.24853

**Published:** 2015-07-21

**Authors:** Petr Rathner, Adriana Rathner, Michaela Horničáková, Christian Wohlschlager, Kousik Chandra, Jaroslava Kohoutová, Rüdiger Ettrich, Reinhard Wimmer, Norbert Müller

**Affiliations:** ^1^ Institute of Organic Chemistry, Johannes Kepler University LinzLinz4040Austria; ^2^ Faculty of Science, University of South BohemiaČeské BudějoviceCzech Republic; ^3^ Lohmann Animal HealthCuxhaven27472Germany; ^4^ Scientific Computing, Johannes Kepler University LinzLinz4040Austria; ^5^ Center of Nanobiology and Structural Biology, Institute of Microbiology, Academy of Sciences of the Czech RepublicNové HradyCzech Republic; ^6^ Department of BiotechnologyChemistry and Environmental Engineering, Aalborg UniversityAalborg9220Denmark

**Keywords:** dynamic N‐terminus, extrinsic photosynthetic protein, hydrogen bond dynamics, intrinsic disorder, solution structure, *Spinacia oleracea*

## Abstract

The extrinsic proteins of photosystem II of higher plants and green algae PsbO, PsbP, PsbQ, and PsbR are essential for stable oxygen production in the oxygen evolving center. In the available X‐ray crystallographic structure of higher plant PsbQ residues S14‐Y33 are missing. Building on the backbone NMR assignment of PsbQ, which includes this “missing link”, we report the extended resonance assignment including side chain atoms. Based on nuclear Overhauser effect spectra a high resolution solution structure of PsbQ with a backbone RMSD of 0.81 Å was obtained from torsion angle dynamics. Within the N‐terminal residues 1–45 the solution structure deviates significantly from the X‐ray crystallographic one, while the four‐helix bundle core found previously is confirmed. A short α‐helix is observed in the solution structure at the location where a β‐strand had been proposed in the earlier crystallographic study. NMR relaxation data and unrestrained molecular dynamics simulations corroborate that the N‐terminal region behaves as a flexible tail with a persistent short local helical secondary structure, while no indications of forming a β‐strand are found. Proteins 2015; 83:1677–1686. © 2015 The Authors. Proteins: Structure, Function, and Bioinformatics Published by Wiley Periodicals, Inc.

***Abbreviations***
OEC: oxygen evolving complex
PSII: Photosystem II
RMSF: root mean square fluctuations

## INTRODUCTION

Photosystem II (PSII) accomplishes arguably the most essential process for aerobic life on Earth, which is the water splitting reaction. The mechanism of oxygen generation has remained conserved during the evolution for more than two billion years.^1^ In higher plants and cyanobacteria PSII consists of several intrinsic membrane‐spanning and a number of extrinsic proteins on the lumenal side of thylakoid. In contrast to the highly conserved intrinsic proteins, the extrinsic photosynthetic proteins forming the oxygen evolving complex (OEC) vary considerably between various photosynthetic organisms. To sustain a high rate of oxygen evolution in higher plants, the extrinsic proteins PsbO (33 kDa), PsbP (23 kDa), PsbQ (16.5 kDa), and PsbR (10 kDa) are essential.^2^, ^3^ In cyanobacteria, the PsbP and PsbQ homologues (denoted as CyanoP and CyanoQ, respectively) are present together with the PsbU and PsbV extrinsic proteins.^4^, ^5^ The main functions of these loosely attached extrinsic proteins are perceived to be the protection of the highly reactive manganese cluster from exogenous reductants and the regulation of the ionic environment.^6^, ^7^ Among the extrinsic proteins of PSII, PsbO is so far the most extensively studied one, a fact probably owed to its ubiquity in all photosynthetic species. Preliminary NMR investigations of PsbO from *T. elongatus* indicated, that PsbO is a folded protein with numerous disordered regions.^8^

Concerning the PsbQ and PsbP proteins, it is supposed that their interactions play a crucial role in the regulation of calcium and chloride ion concentrations within the PSII complex.^9^ Recent studies showed that PsbQ can compensate for a common structural defect of PsbP, that is, truncation of the 15 N‐terminal residues which are required for binding to PSII. PsbQ thus contributes to the protection of the catalytic manganese cluster.^10^ It has been shown that the PsbP and PsbQ proteins may also directly associate with PSII intrinsic subunits.^11^ High resolution crystal structures of PsbP and PsbQ from higher plants^12^, ^13^, ^14^, ^15^ and cyanobacteria^16^ have been reported. In all crystallographic structures of PsbP and PsbQ, including Psb31 from diatom algae, significant parts of the sequences are missing.^12^, ^13^, ^14^, ^15^, ^16^, ^17^ This applies also to the PsbQ protein from *Spinacia oleracea* (spinach). A previous crystallographic study of spinach PsbQ found a well‐defined C‐terminal four‐helix‐bundle and a loosely packed N‐terminus, from which 20 residues (S14‐Y33) are missing.^14^ Recent cross‐linking experiments of PsbP and PsbQ proteins revealed a close interaction of K176 in PsbP with D28 in PsbQ.^18^ Since this cross‐linked site of PsbQ is not visible in the electron density map, it was supposed that the N‐terminal stretch of free PsbQ is very flexible and probably extended in solution.^14^ Previous NMR studies of the secondary structure of PsbQ in solution indicated some residual order in the so far unresolved dynamic N‐terminal region^19^ which was however inconsistent with the short parallel β‐sheet (I5‐V7 and F38‐L40) reported in the crystallographic structure.^14^

In this article, we present the nearly complete ^1^H, ^15^N and ^13^C backbone and side‐chain NMR assignments of recombinant PsbQ from *Spinacia oleracea* together with its high resolution NMR structure in solution. Supporting Information obtained from ^15^N NMR relaxation, {^1^H}^15^N NOE experiments and unrestrained MD simulations yielded first information on internal backbone dynamics. The N‐terminal segment, which apparently is essential for the interactions of PsbQ with other constituents at the lumenal side of the thylakoid near PSII, is a main point of interest in this investigation.

## MATERIALS AND METHODS

### Protein preparation

Cloning, overexpression and purification of uniformly doubly labeled ^15^N, ^13^C PsbQ from *Spinacia oleracea* in *E. coli* were described previously.^19^ The samples for NMR spectroscopy contained 800 µM ^15^N,^13^C PsbQ, 20 m*M* Na_2_HPO_4_, 50 µM NaN_3_, 1 m*M* EDTA (pH = 7.0) in H_2_O: ^2^H_2_O 90:10.

### NMR spectroscopy

All NMR experiments were performed on 700 MHz Bruker Avance III spectrometer equipped with TCI cryogenically cooled probe (20 K) at 298 K sample temperature. HC(C)H‐TOCSY,^20^ (H)C(C)(CO)NH,^21^ HBHA(CO)NH,^22^ HC(C)H‐COSY,^23^, ^24^, ^25^ TOCSY‐HSQC,^26^ (HB)CB(CGCD)HD and (HB)CB(CGCDCE)HE^27^ experiments were used to complement the proton and carbon shifts published previously (BMRB entry 17357).^19^ The improved and extended set of assignments has been submitted to BMRB (entry 25350). To obtain information about the internal dynamics of PsbQ, ^15^N backbone relaxation experiments were investigated. ^15^N‐T_1_ and ^15^N‐T_2_ relaxation rates^28^ were computed by exponential fitting of peak heights in sets of different ^15^N‐HSQC phase sensitive experiments with different relaxation delays. T_1_ relaxation delays: 10, 50, 100, 200, 300, 400, 500, 600, 700, 800, 900, 1000, 1100, and 1200 ms. T_2_ relaxations delays: 10,15, 20, 25, 35, 40, 50, 60, 70, 80, 90, 100, and 120 ms. The backbone {^1^H}^15^N NOEs were calculated from ratios of peak heights from two heteronuclear NOESY ^15^N‐^1^H sensitivity enhanced HSQC‐type experiments.^28^

Subsequently, three‐dimensional ^15^N‐edited (mixing time 60 ms) and ^13^C‐edited (aliphatic and aromatic, mixing time 100 ms) NOESY‐HSQC experiments^26^, ^29^, ^30^ were involved to obtain distance restrains. All NMR data were recorded and processed with Bruker Topspin software (v. 3.1 and 3.2). Spectra analysis including resonance assignment was carried out manually with the help of CARA software.^31^

### Structure calculation

NOESY cross‐peaks were integrated by the NEASY subroutine^32^ of CARA and converted into upper distance constraints using the CALIBA subroutine.^33^ Dihedral angle constraints were estimated from backbone chemical shifts using TALOS‐N.^34^ ϕ and ψ angles of residues whose multiple‐database prediction did not match within the same region of the Ramachandran map, were discarded. Upper‐distance‐ as well as dihedral‐angle‐constraints were used as input for the structure calculations using the torsion angle dynamics program CYANA (v. 3.0).^35^ In total 100 structures were calculated in 10,000 annealing steps. The 20 conformers with lowest CYANA target function values were refined in a water box by restrained MD simulations using the YASARA force field^36^ implementation in YASARA 12.1.19.^37^ The atomic coordinates of the refined bundle of 20 conformers shown in Figures 2 and 3(A) have been deposited in the protein database under PDB ID: 2MWQ.

### Molecular dynamics simulations

As a major part of the N‐terminal sequence is unresolved in the X‐ray structure of PsbQ, an earlier reported model^38^ based on the X‐ray structure and loop modelling has been used as representative for the X‐ray structure. Of the NMR structures a conformer out of the middle of the conformational ensemble was selected to represent the NMR structure. GROMACS 4.6.3 package^39^, ^40^, ^41^ was used for preparing the system and performing MD simulations using the Amber99 SB force field.^42^ Both protein structures were solvated by explicit TIP3P water^43^ in a cubic box with a margin of 10 Å between solute and the box walls. Systems were neutralized by addition of sodium counter ions. The particle‐mesh Ewald method^44^ was applied to calculate long‐range electrostatic interactions with a cut‐off distance of 10 Å. A Lennard‐Jones 6–12 potential was used to evaluate van der Waals interactions within 10 Å cut‐off distance. The LINCS algorithm of fourth order expansion was used to constrain bond lengths.^45^ After solvation and neutralization steps the system was optimized for 10000 steps using the steepest‐descent method to remove steric clashes between atoms. The system was equilibrated for 1 ns with position restraints of 1000 kJ/mol on all heavy atoms. A constant temperature of 300 K or 310 K was maintained using the V‐rescale algorithm^46^ with a coupling time of 0.1 ps and separate baths for the solute and the solvent. The pressure was kept constant at 1 bar using the Parrinello‐Rahman pressure coupling scheme^47^ with a time constant of 2 ps. Initial velocities were generated randomly using a Maxwell‐Boltzmann distribution corresponding to 300 K and additionally for 310 K in case of the X‐ray structural model. Neighbour lists were updated every 10 fs using a group cut‐off scheme. Finally the production runs were performed in the isothermal‐isobaric (NPT) ensemble without restraints for 225 ns for the X‐ray structure at 300 K and 310 K, and for 300 ns for the NMR‐structure at 300 K. The grmf tool of the GROMACS package was used to calculate root mean square fluctuations (RMSF) during the last 20 ns of trajectories.

VMD^48^ was used to calculate interatomic distances and for manual inspection of trajectories. We employed xmgrace (http://plasma‐gate.weizmann.ac.il/Grace/) for pre paring the graphs.

## RESULTS AND DISCUSSION

### NMR assignment

**Figure 1:**
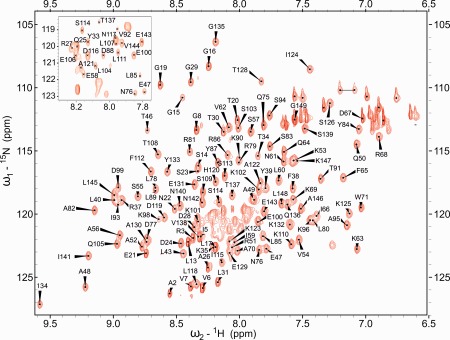
2D [^1^H‐^15^N]‐HSQC spectrum of 0.8 m*M* ^15^N,^13^C‐PsbQ in 20 m*M* KH_2_PO_4_, 1 m*M* EDTA, pH = 7.0 (H_2_O:D_2_O 90:10 v/v), *T* = 298 K.

Employing the set of experiments summarized in the section on NMR spectroscopy, the resonance assignments (BMRB entry 17357) of PsbQ from *Spinacia oleracea* were extended and improved. In total 718 of 739 backbone (97.2%) and 839 of 1173 (71.5%) side chain resonance chemical shifts were assigned as compared to 80.7% and 40.1% of backbone and side chain resonances in the earlier assignment,^19^ respectively. The backbone amide resonances of five non‐proline amide residues (E1, D36, Q41, S73, and L74) could not be identified or assigned unequivocally due to fast NH exchange and/or severe peak overlap in the ^15^N‐HSQC (Fig. 1). <30% of the side‐chain resonances remained unassigned due to signal overlap in HC(C)H‐COSY and TOCSY‐HSQC spectra.

### Restraints, structure calculation, and analysis

Through assignment of the cross‐peaks in the ^15^N‐, ^13^C_ali_‐ and ^13^C_aro_‐NOESY‐HSQC experiments, typical α‐helical NOE patterns [*d*_NN_(*i*,*i* + 1), *d*_αN_(*i*,*i* + 1), *d*_βN_(*i*,*i* + 1), and *d*_αN_(*i*,*i* + 3)] were found in all helices proposed in our previous study^19^ largely coinciding with the helix‐bundle identified by X‐ray crystallography.^14^ Only a very limited number of NOE cross‐peaks could be found involving the first 35 residues, presumably due to the high mobility of the N‐terminus. The majority of NOE constraints was found in the well‐defined C‐terminal region of the molecule, mainly from the ^15^N‐NOESY‐HSQC spectrum. 177 long range NOE cross‐peaks (|*i*−*j*|≥5) were assigned. Note that PsbQ contains 13 proline residues, which lack amide protons. In total, 872 experimentally derived upper distance limits and 187 angular constraints were used as input information for the CYANA structure calculations. Subsequent rMD simulations using the YASARA force field^36^ yielded a final set of 20 conformers without any experimental distance violation larger than 0.1 Å. The ensuing structural statistics are summarized in Table 1.

**Table 1:** Structural Statistics for the Energy‐Minimized NMR Solution Structure of PsbQ

Experimental constraints	
NOE based distance constraints	
Total	872
Intra‐residue [*i* = *j*]	262
Sequential [|*i* − *j*| = 1]	315
Medium range: [1<|*i* − *j*|<5]	118
Long range: [|*i* − *j*|≥5]	177
NOE constraints per restrained residue	6.3
Dihedral‐angle constraints^a^:	187
Total number of restricting constraints	1059
Total number of restricting constraints per restrained residue	7.6
Violations	
Max. distance violation (Å)	0.10
RMS of distance violation/constraint (Å)	0.01
Maximal torsion angle constraint violation (°)	5.00
RMS of dihedral angle violation/structure (°)	0.19
Structure quality	
CYANA target function value (Å^2^)^b^	2.24 ± 0.31
Backbone [C^α^, C′, N] RMSD from average (Å)^c^	0.81 ± 0.15
Heavy atoms RMSD from average (Å)^c^	1.63 ± 0.20
Total energy (kJ·mol^−1^)	−54429.0 ± 521.2
Ramachandran plot summary for selected residues^d^ from Procheck^52^	
Most favored regions	95.9%
Additionally allowed regions	4.0%
Generously allowed regions	0.0%
Disallowed regions	0.0%

a Derived from backbone chemical shifts by Talos‐N.^34^

b Before water refinement.

c Calculated for non‐mobileresidues (45–149) after water refinement.

d Residues 9–11, 37–95, 99–148.

**Figure 2:**
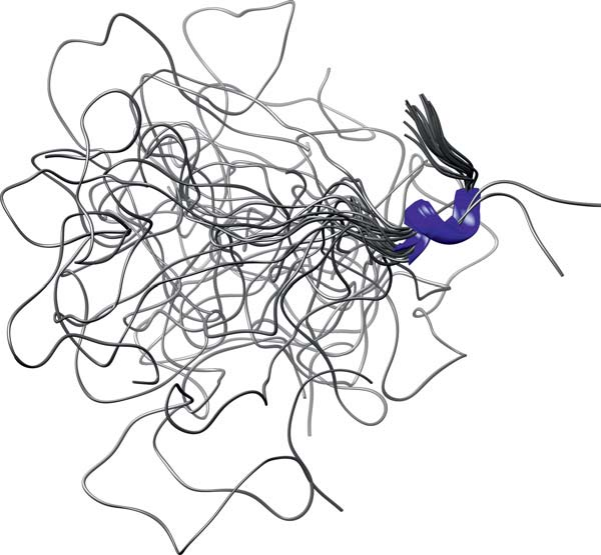
Superimposed backbone structure representations of the N‐terminal regions (E1‐P45) of 20 water refined conformers aligned at the α helix 0 (R37‐L40).

The backbone (C^α^, C′, N) RMSD for the region of the four α‐helices (residues 46–149) is 0.81 ± 0.15 Å, while for the N‐terminal part (residues 1–36) we find only an RMSD of 7.74 ± 1.50 Å. However, a short α‐helix, which we call 0 remains persistent among the 20 low energy structures (Fig. 2). The backbone (C^α^, C′, N) RMSD for these four residues is 0.24 ± 0.13 Å.

**Figure 3:**
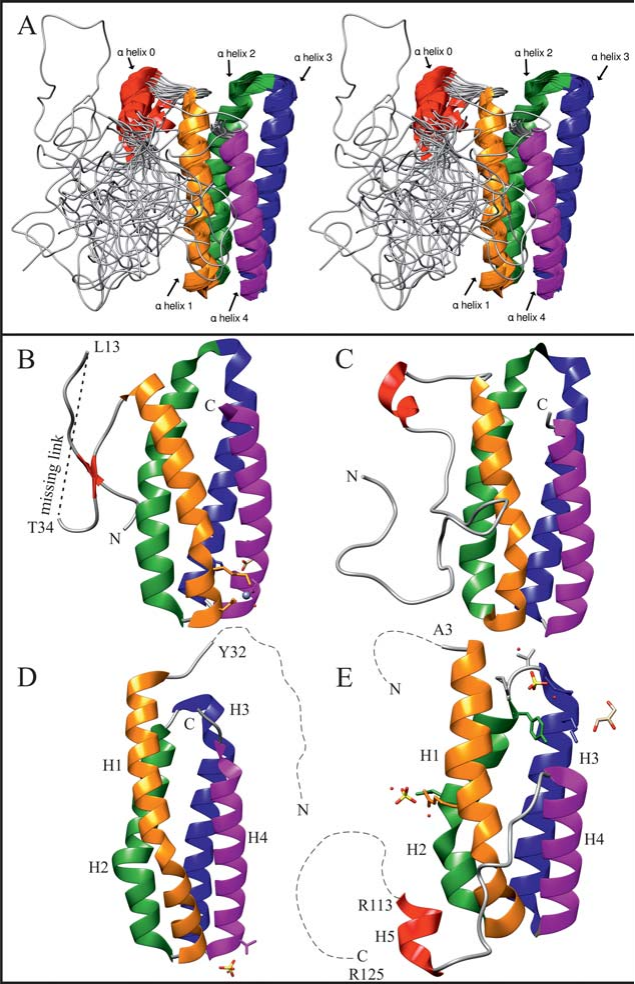
(A) Stereoscopic ribbon representation of 20 spatially aligned (at the region of amino acid residues 45–149) water refined conformers. (**B**) Crystal structure of spinach PsbQ.^14^ (**C**) NMR solution structure of spinach PsbQ with the lowest restraint violation energy. (**D**) Crystal structure of PsbQ from *Synechocystis* sp. PCC 6803.^16^ (**E**) Crystal structure of diatom Psb31.^17^ The dashed lines represent unresolved regions. The figure was produced using UCSF Chimera.^51^

In summary, the solution structure of PsbQ from *Spinacia oleracea* can be described as belonging to an all‐α protein class with a bundle of four up‐down‐up‐down α‐helices and a flexible N‐terminus [Fig. 3(A)]. In comparison with the most up‐to‐date available crystallographic structure (PDB: 1VYK), the larger C‐terminal part (residues 46‐149) corresponding to the four‐helix bundle remains largely conserved in the mean solution structure with a backbone (C^α^, C′, N) RMSD of 1.27 Å with respect to the X‐ray structure, while the N‐terminus emerges as predominantly unfolded, with one important exception. Based on the experimental NOE data, residues 37–40 located in the dynamic N‐terminus of the molecule exhibit high α‐helical character. Remarkably, this is exactly at the location where one of the two short β‐strands had been proposed in the crystal structure [Fig. 3(B)]^14^ and by Raman spectroscopy.^38^ No indication of any well‐defined secondary structure could be found for residues 5–7 where the complementary β‐strand was reported in the X‐ray structure. In the crystallographic structure of a related cyanobacterial protein (PsbQ from *Synechocystis sp*. PCC 6803)^16^ [Fig. 3(D)] helix H1, corresponding to α‐helix 1 in our structure, is extended toward the N‐terminus but ends short of the location of the short helix 0 in the solution structure of spinach PsbQ. In the Supporting Information the experimental NOE‐patterns in this range are compared to predictions of NOE patterns corresponding to the X‐ray structure, clearly corroborating the existence of this α‐helix. Interestingly, the NMR solution structure appears to be very similar to the crystallographic structure of Psb31 from the diatom *Chaetoceros gracilis*, although the sequence similarity between Psb31 and spinach PsbQ, compared with other PsbQ‐like proteins, is very low (25.5%).^17^ However, while PsbQ has a flexible N‐terminal stretch with an embedded short α‐helix, Psb31 exhibits a similar feature on the C‐terminus [Fig. 3(E)].

### Backbone atom relaxation studies

**Figure 4:**
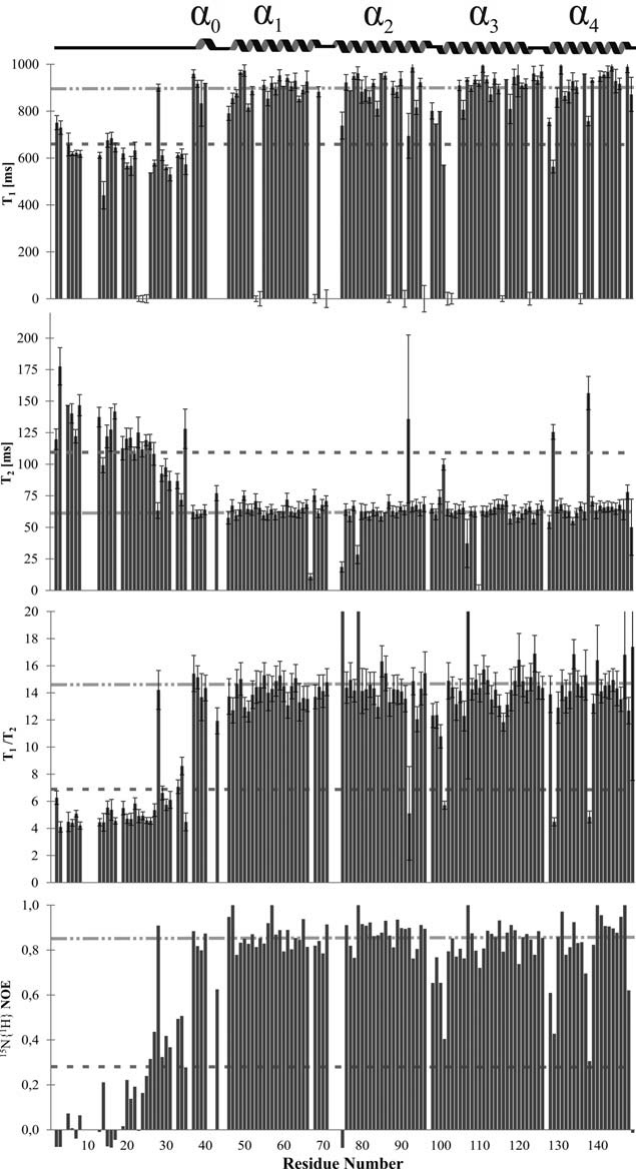
Heteronuclear {^1^H}^15^N NOEs enhancements, ^15^N T_1_ and ^15^N T_2_ relaxation rates of PsbQ. The NOE enhancements show small values for residues 2–35 indicating high backbone flexibility in this region. Notably, the values for residues 37–41 are higher, indicating conformational stability of the short α helix 0 [Fig. 3(A)] found by the homonuclear NOE experiments. Correspondingly, the *T*_1_ and *T*_2_ values indicate the reduced mobility of residues 37–41 and the dynamic nature of the conformation within the first 35 residues. The heteronuclear NOEs and relaxation rates confirm rigidity of the four‐helix bundle in Figure 3(A).

Since the current and previous experimental studies have indicated high internal mobility of the N‐terminal segment of PsbQ, we used dynamic NMR methods ^15^N longitudinal and transverse relaxation times *T*_1_, *T*_2_, respectively, and the heteronuclear {^1^H}^15^N NOEs for backbone dynamics analysis summarized in Figure 4. The backbone ^15^N T_1_, and T_2_ relaxation times as well as the {^1^H}^15^N NOEs data corroborate the mobility information obtained in the experimental ^15^N and ^13^C filtered ^1^H‐^1^H NOE, apart from a few outliers. For several residues including the 13 prolines (P4, P9, P10, P11, P12, P18, P32, P42, P44, P45, P72, P97, P127) and the 6 unassigned residues' (E1, D36, Q41, D67, S73, L74) {^1^H}^15^N NOEs and relaxation data could not be determined. The averaged T_1_/T_2_ ratio over all measured residues was 12.6 ± 5.1, which is in the expected range for a near globular protein of this size. By contrast, the average *T*_1_/*T*_2_ ratio for the residues 2–35 was found to be 6.92 ± 3.6. The *T*_1_/*T*_2_ ratios rapidly increase in the well‐defined C‐terminal helical bundle region including the residues of the short α‐helix 0 (R37‐Q41). The overall rotational correlation time *τ*_c_ for the four‐helix bundle (residues 46–149) of PsbQ was calculated^49^ as 9.91 ± 1.14 ns (298 K). Complementary {^1^H}^15^N NOE data also prove the high mobility of the first 35 residues. By comparison to the average of the first 45 residues, the numbers of NOE cross‐peaks found for all helical parts are significantly higher (Fig. 4).

The heteronuclear NOEs and relaxation rates corroborate the first 35 residues of the N‐terminal region and the residues between helix 0 and helix 1 to be highly flexible and intrinsically disordered. The presence of a polyproline II helix (residues 9–12) suggested in the crystallographic structure^14^ could not be confirmed by NMR in solution. The heteronuclear {^1^H}^15^N NOEs, ^15^N T_1_ and ^15^N T_2_ relaxation rates of the immediately adjacent residues (V6‐G8, L13‐G15) contradict the notion of any ordered conformation in this region.

### Molecular dynamics simulations

**Figure 5:**
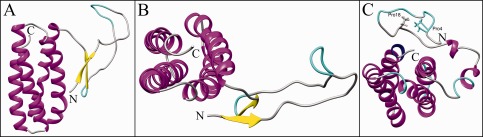
Simulated PsbQ structures after unrestrained molecular dynamics in solvent. (**A**) Side view of the X‐ray‐based model after 225 ns at 300 K. Helices are coloured in magenta, β‐strands in yellow and β‐turn structures in cyan. 3_10_ helices are drawn in blue. N and C terminals are labelled accordingly. (**B**) Top view of the structure in panel A. (**C**) Top view of the NMR structure after 300 ns at 300 K.

**Figure 6:**
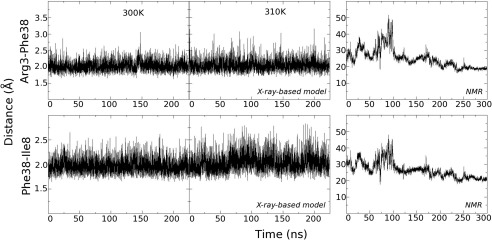
Dynamics of hydrogen‐bonding between the two N‐terminal β‐strands. Left and middle panels: Distances between Phe38‐H Arg3‐O (upper panels) and Phe38‐O Ile5‐H (lower panels) in the X‐ray based model structure in the course of the MD simulations at 300 K and 310 K. Right panels: Distances between Phe38‐H Arg3‐O (upper panel) and Phe38‐O Ile5‐H (lower panel) in the MD simulation of the NMR structure.

**Figure 7:**
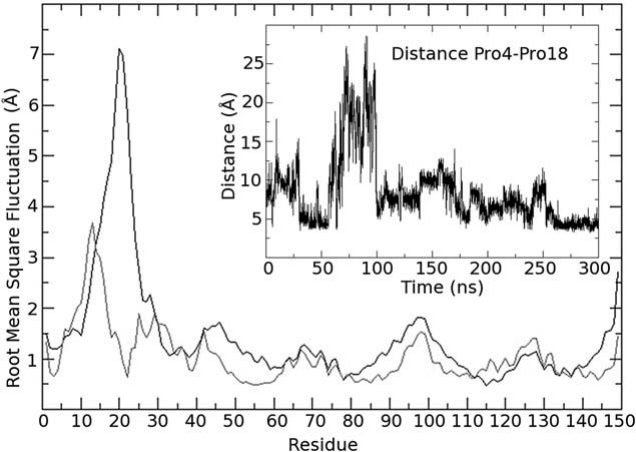
RMSF of PsbQ during the last 20 ns of MD simulation at 300 K. Black curve: Starting from the X‐ray‐based model structure, grey curve: Starting from the NMR structure. The insert shows the distance between the gamma carbons of Pro4 and Pro18 in the course of the MD simulation of the NMR structure.

Earlier work based on the X‐ray crystal structure^38^ suggested that the two‐stranded β‐sheet would anchor the large N‐terminal loop between Leu13 and Thr34 and weak interactions with the rest of the protein would give this loop a random coil structure with a distinct stable fold. The herein reported NMR‐results are directly contradicting this hypothesis and therefore new molecular dynamics simulations were set up for both structures, the X‐ray‐based model structure used in the earlier study, and the newly reported NMR structure. As the above mentioned conclusions were drawn from 20 ns long simulations, that were state‐of‐the‐art then, longer simulations, which are possible nowadays, might show that the conformation the loop adapted could have been only a local minimum and on longer time scales might sample a much larger conformational space. For the X‐ray‐based model a slightly elevated temperature of 310 K was used additionally to allow for possible jumping over an energy barrier, if the protein would be trapped in a local minimum. Equilibration of the simulations was estimated by root mean square deviation (not shown) and sufficiently long trajectories were calculated to have at least the last 30 ns in equilibrium. Thus, simulations were performed for 225 ns or 300 ns, respectively. Figure 5 shows the X‐ray‐based crystal structure after 225 ns and the NMR structure after 300 ns of molecular dynamics simulation at 300K in water, and surprisingly the two stranded β‐sheet in the X‐ray‐based model kept its secondary structure. The two hydrogen bonds between Phe38‐H Arg3‐O and Phe38‐O Ile5‐H that keep the two β‐strands together are persistent throughout the simulations and show only slightly larger fluctuations at elevated temperature of 310K (Fig. 6, left and middle panels). However, Figure 6, left panel, also demonstrates that the loop structure has significantly changed from the initial structure and that there are no weak contacts between the loop and the helix‐bundle of the protein left over, but the loop is rather oriented toward the solvent. In the simulation of the NMR structure, the respective residues get never closer than 20 Å and thus there is no possibility to form hydrogen bonds or the β‐sheet (Fig. 6, right panels). Contrary to this, the NMR solution structure shows weak interactions between Pro4 and Pro18, manifest by the distance between the gamma carbons of both proline residues in the insert of Figure 7. The RMSF during the last 20 ns of the simulations, in which both simulations are well equilibrated, show extremely high values of up to 7 Å for the N‐terminal loop (Fig. 7, black line) in the X‐ray‐based model, while the NMR structure shows fluctuations only slightly higher than for the loops connecting the helices in the bundle (Fig. 7, gray line). Although the β‐sheet is still present in the X‐ray‐based model after the simulation, this clearly contradicts the original hypothesis that the β‐sheet would anchor the loop in a specific conformation. The loop in the simulations does not keep any weak interactions with the rest of the protein and does not show a distinct stable fold, but is extremely flexible, giving the impression of unsuccessfully sampling the conformational space for a local minimum. In contrast to that, the loop in the simulations with the NMR structure rearranges in the course of the simulation and finally keeps a metastable distinct fold with comparably low fluctuation. There is no indication in these simulations that the N‐terminal would preferentially form a β‐strand. On the contrary, the helical structure has been shown to be stable throughout the simulation. This would be consistent with an understanding that the N‐terminal, if not complexed by PSII, is rather unstructured and behaves as a tail, sampling a large conformational space with various local minima. This is also supported by the fact that in the recent crystal structure of CyanoQ^50^ the first 34 amino acids are not resolved as well, in spite of a generally high resolution of 1.6 Å.

## CONCLUSION

In this article we presented the solution structure of the accessory photosynthetic protein PsbQ from *Spinacia oleracea* based on the near complete resonance assignment. Using 3D NMR experiments at 700 MHz on recombinant, uniformly ^13^C, ^15^N labeled PsbQ, the resonance assignment levels of 97.2% of the backbone and 71.5% of the side chain residues were achieved. Nuclear Overhauser effect based distance constraints and chemical shift derived torsion angle constraints led to a structure ensemble with backbone (C^α^, C′, N) RMSD of 0.81 ± 0.15 Å for the well‐defined four‐helix‐bundle region. A highly flexible N‐terminal region is attached to this core. The bundle of four up‐down‐up‐down α‐helices corresponds closely to the previous crystallographic structure^14^ with a backbone (C^α^, C′, N) RMSD of 1.27 Å with respect to the mean NMR structure. However, a short α‐helix within residues R37‐L40 is found in a region, where one half of the two‐stranded β‐sheet had been derived previously in the crystal structure.^14^ It is noteworthy that D28, which has been found to interact with PsbP in a cross‐linking study,^18^ is part of the flexible tail preceding the short helix. It is thus able to access the large range of orientations, which may be a requirement for the repair functions of PsbQ for the OEC (for example, under redox stress conditions) suggested in a previous study.^10^ The unrestrained molecular dynamics simulations for up to 300 ns in water support the understanding that in solution and in absence of other interaction partners the N‐terminal region behaves as a flexible tail with an embedded conserved short α‐helix rather than being anchored by a two strand β‐sheet. This short helix might play a role in the assembly process of the OEC lumenal protein complex. In the fully assembled PSII complex, the N‐terminal part could not only form either of the two proposed structures but assume a different conformation induced by intermolecular interactions. It is noteworthy that in the recently determined crystal structure of the Psb31 protein from *Chaetoceros gracilis*, which shows very low sequence homology to PsbQ but seem to be a functional equivalent, a similar global structure, a four‐helix‐bundle with a flexible tail with an embedded short α‐helix which, by contrast, is attached at the C‐terminal side has been found.^17^ This may be an indication of both intrinsically disordered regions performing similar maintenance functions.

While flexible terminal regions are quite common in protein structures it appears from biological evidence that they have very determined roles within the Psb proteins.^10^ The experimental NMR and computational MD results reported thus corroborate that the flexible, intrinsically disordered, regions of the Psb proteins of higher plant PSII and in particular of PsbQ play a key role in protein‐protein interactions. Their solution structures and dynamics appear to be essential for understanding the assembly of the OEC and the functions of the extrinsic PSII proteins. The roles of the unstructured parts including the long flexible tails in the Psb protein interactions may be multiple, as is typical for intrinsically disordered regions. They may very likely adopt different conformations and exhibit different dynamics during the assembly process of the OEC, regulation of ion concentration while anchored to the thylakoid, and when performing the aforementioned repair function,^10^ respectively. Based on the current results and the recent NMR assignment of PsbP,^53^ which both are probably most relevant for the assembly stage, our future investigations will include all extrinsic Psb proteins of the OEC found in higher plants. Exploring their mutual interactions as well as the influence of the ionic and membrane environment on their structural and dynamic features will reveal their functions in greater detail.

## Supporting information

Supporting Information
