# Supplementary material for: Solution NMR and molecular dynamics reveal a persistent alpha helix within the dynamic region of PsbQ from photosystem II of higher plants
Source: Proteins. 2015 Jul 21;83(9):1677–86. doi: 10.1002/prot.24853 (PMC4758407; doi:10.1002/prot.24853)
Supplement: Supplementary file 1 — Supporting Information [file PROT-83-1677-s001.pdf]

Supplementary Material

The experimental and predicted NOE-patterns in the sequence regions of α-helix 0 in the solution structure and the beta sheet in the X-ray-structure of PsbQ[38] are compared in Fig. S1. The experimental NOE pattern is only compatible with an α-helical structure for residues 37-40.

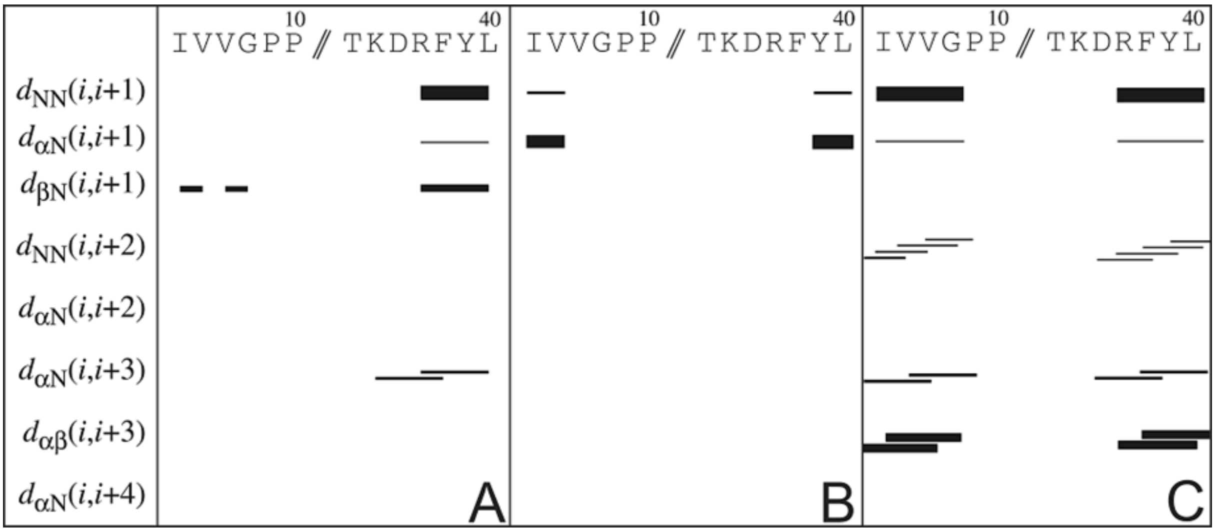

Figure S1: Experimental NOE-patterns (A) compared to predicted NOE patterns for the ß-sheet structure (B) found in the X-ray study[38] or for assumed pure α-helices (C) in place of these two ß-strands.
